# Genome-wide association analyses of carcass traits using copy number variants and raw intensity values of single nucleotide polymorphisms in cattle

**DOI:** 10.1186/s12864-021-08075-2

**Published:** 2021-10-23

**Authors:** Pierce Rafter, Isobel Claire Gormley, Deirdre Purfield, Andrew C. Parnell, Saeid Naderi, Donagh P. Berry

**Affiliations:** 1 Animal & Grassland Research and Innovation Centre, Teagasc, Moorepark, Cork Fermoy, Ireland; 2 School of Mathematics and Statistics, University College Dublin, Belfield, Dublin 4, Ireland; 3 Department of Biological Sciences, Munster Technological University Institute, Cork Bishopstown, Ireland; 4 Hamilton Institute, Insight Centre for Data Analytics, Maynooth University, Kildare, Ireland; 5 Irish Cattle Breeding Federation, Cork Bandon, Ireland

**Keywords:** CNV, Log R ratio, Holstein-Friesian, Charolais, Limousin, Fluorescence intensity

## Abstract

**Background:**

The carcass value of cattle is a function of carcass weight and quality. Given the economic importance of carcass merit to producers, it is routinely included in beef breeding objectives. A detailed understanding of the genetic variants that contribute to carcass merit is useful to maximize the efficiency of breeding for improved carcass merit. The objectives of the present study were two-fold: firstly, to perform genome-wide association analyses of carcass weight, carcass conformation, and carcass fat using copy number variant (CNV) data in a population of 923 Holstein-Friesian, 945 Charolais, and 974 Limousin bulls; and secondly to perform separate association analyses of carcass traits on the same population of cattle using the Log R ratio (LRR) values of 712,555 single nucleotide polymorphisms (SNPs). The LRR value of a SNP is a measure of the signal intensity of the SNP generated during the genotyping process.

**Results:**

A total of 13,969, 3,954, and 2,805 detected CNVs were tested for association with the three carcass traits for the Holstein-Friesian, Charolais, and Limousin, respectively. The copy number of 16 CNVs and the LRR of 34 SNPs were associated with at least one of the three carcass traits in at least one of the three cattle breeds. With the exception of three SNPs, none of the quantitative trait loci detected in the CNV association analyses or the SNP LRR association analyses were also detected using traditional association analyses based on SNP allele counts. Many of the CNVs and SNPs associated with the carcass traits were located near genes related to the structure and function of the spliceosome and the ribosome; in particular, *U6* which encodes a spliceosomal subunit and *5S rRNA* which encodes a ribosomal subunit.

**Conclusions:**

The present study demonstrates that CNV data and SNP LRR data can be used to detect genomic regions associated with carcass traits in cattle providing information on quantitative trait loci over and above those detected using just SNP allele counts, as is the approach typically employed in genome-wide association analyses.

**Supplementary Information:**

The online version contains supplementary material available at 10.1186/s12864-021-08075-2.

## Background

The carcass value of cattle in most jurisdictions is a function of the carcass weight, with additional premia paid for animals that meet particular specifications on age, breed-type, and certain carcass conformation and carcass fat metrics [1, 2]. The heritability of carcass traits is estimated to be in the range of 0.30 to 0.40 [3–5], signifying the presence of genetic variation. Many studies in cattle have attempted to locate quantitative trait loci (QTLs) associated with this genetic variation. These studies have generally limited their investigation to bi-allelic single nucleotide polymorphism (SNP) allele count data [6–8]. Similarly, genomic association studies of other traits in cattle, such as milk and fertility traits [9, 10], as well as association analyses in other domestic livestock species [11], tend to limit the independent variable in their analyses to the allele count for a given SNP. However, other types of genetic variants such as copy number variants (CNVs) exist; these genetic variants are formed by duplication or deletion of segments of DNA [12]. Copy number variants are typically considered to have a minimum length between 50 bp [13] and 1 kb [12] depending on the method used to detect the CNVs. Previous imputation studies indicate that CNVs may not be in strong linkage disequilibrium with flanking SNP alleles [14, 15]. Xu et al. [16] performed association analyses between CNVs and milk traits in Holsteins; approximately 25 % of the associated CNVs they identified were not in linkage disequilibrium with flanking SNPs. Therefore, association studies that also include CNV data as well as SNP allele data may reveal additional QTLs associated with a trait of interest, which otherwise might not be discoverable using SNP allele count data alone. Genome-wide association analyses in cattle using CNV data are limited in number, but those that exist have identified CNVs associated with meat tenderness in a population of 723 Nelore cattle [17], carcass and growth traits in a population of 2,230 Nelore cattle [18], milk performance in a population of 1,116 Brown Swiss cattle [19], and somatic cell count in 242 Holstein cows [20].

One of the key metrics used to detect CNVs from SNP array data is the normalized fluorescence intensity of the SNP. The fluorescence intensity reported on Illumina genotype platforms is measured by the log R ratio (LRR) value, where the LRR value of a SNP is the log_2_ of the observed fluorescence intensity of the SNP divided by the expected fluorescence intensity of the SNP [21]. The expected fluorescence intensity of the SNP is calculated using linear interpolation between the genotype clusters of the SNP [22]. To the best of our knowledge no previous genome-wide association analyses in cattle have directly used SNP LRR information, although Salomon-Torres et al. [23] used the normalized fluorescence intensity values of SNPs in a cluster analysis to identify Holsteins that are at risk of ovarian pathologies. Jenkins et al. [24] did, however, detect an association between SNP LRR and carcass merit in Romney, Perendale, Coopworth, and Texel New Zealand sheep.

The objectives of the present study were two-fold: firstly, to perform association analyses of carcass traits in cattle using CNV copy number, and secondly to perform separate association analyses of carcass traits on the same population of cattle but using SNP LRR values. Of interest for both of these objectives was whether or not any discovered associations could also be detected using only the allele count data for individual SNPs, as this is the approach most commonly undertaken in genome-wide association analyses.

## Results

Four different association analyses were carried out in the present study, with the models applied differing only by the independent variable in the model. The four models evaluated included each carcass trait as the dependent variable but the independent variable was either the CNV copy number for a given detected CNV, the SNP LRR per SNP or, at a chromosomal level, the proportion of the chromosome with a called CNV or the mean SNP LRR per chromosome. The single CNV and SNP LRR association analyses were used to identify individual CNVs and SNP LRRs associated with carcass traits while both the CNV proportion and mean LRR per chromosome analyses were used to determine if overall CNV burden, or mean LRR as a metric possibly reflective of CNV burden, associated with carcass merit.

### Chromosome association analyses

The proportion of the chromosome with a called CNV (either deletion or duplication) did not associate with any of the carcass traits in any of the three breeds. The proportion of chromosome 20 with a called duplication CNV was negatively associated (*P* = 0.0002) with carcass conformation in the Holstein-Friesians; with this exception, there was no other detected association with the proportion of the chromosome with a called duplication CNV or the proportion of the chromosome with a called deletion CNV. The mean LRR of chromosome 13 was positively associated with carcass conformation in Holstein-Friesians (*P* = 0.0006); the regression coefficient for the relationship between carcass conformation and mean LRR of chromosome 13 was 1.059 (SE = 0.307). The mean LRR of all other chromosomes was not associated with any of the carcass traits in any breed.

### Copy number variant association analysis

To be included in the final dataset of CNVs for each breed, the CNV had to be present in at least 3 animals within the breed. After edits, 13,969, 3,954, and 2,805 CNVs were considered for the Holstein-Friesian, Charolais, and Limousin populations, respectively. The population structure of the Holstein-Friesians was less homogeneous than the other two breeds; this was determined from eigen decomposition of the genomic relationship matrices for each of the three breeds. The top eigenvalue for the Holstein-Friesians was approximately 3 times larger than the top eigenvalue for either the Charolais or the Limousins, which indicates that the Holstein-Friesians were less homogeneous than either the Charolais or the Limousins. This may account for the greater number of CNVs available for the Holstein-Friesians than for the Charolais or the Limousins.

Out of the 13,969, 3,954, and 2,805 CNVs tested in the Holstein-Friesian, Charolais, and Limousin cattle, respectively, a total of 16 different CNVs were associated with at least one of the carcass traits in at least one of the three breeds. The number of CNVs tested and the number of associated CNVs for each breed and trait are given in Table 1. One of the CNVs located on chromosome 25 at 15.58 Mb was associated with all three carcass traits in the Holstein-Friesians. All of the remaining CNVs were associated with only one trait and these associations were always only in one breed. Furthermore, there was no overlap in the genomic position of the 16 different associated CNVs. For each associated CNV, the pedigree relationship between animals with the CNV was investigated further to determine if there was an obvious underlying population structure among the animals with that CNV. The purpose of this was to identify associations that may be an artefact of population structure, rather than a true association between the CNV and the carcass trait. For 5 of the 16 associated CNVs, more than half of the animals with the CNV were first or second degree relatives. For each of these 5 CNVs, a power analysis revealed that there was an insufficient number to animals to test if there was a difference in mean deregressed EBV between the half-siblings with the CNV versus the half-siblings without the CNV. These 5 CNVs are presented separately in Table 2; the remaining 11 CNVs which did not have an obvious underlying population structure among the animals with the CNV are presented in Table 3. The marginal R^2^, which is the proportion of the variance attributable to the fixed effect, for each of the associated CNVs was between 0.0002 and 0.0337 (see Tables 2 and 3). Manhattan plots for the CNVs tested against carcass weight, carcass fat, and carcass conformation for the three breeds are given in supplemental Figures S1, S2 and S3, respectively.

**Table 1:** Number of copy number variants (CNVs) available for each breed and trait, and the number of CNVs which were associated (*P* < 0.05) with carcass traits within breed

Breed	Trait	Number of animals	Total number of CNVs	Associated CNVs
Charolais	Weight	945	3,954	1
	Conformation	945	3,954	0
	Fat	945	3,954	0
Holstein-Friesian	Weight	892	13,899	5
	Conformation	915	13,953	8
	Fat	923	13,969	1
Limousin	Weight	974	2,805	0
	Conformation	973	2,804	1
	Fat	974	2,805	2

**Table 2:** Copy number variants (CNVs) associated with each of the carcass traits in Charolais (CH), Holstein-Friesian (HF), and Limousin (LM), but with a possible underlying population structure between the animals with the CNV. When a CNV was present as both duplications and deletions in the population the CNV type was reported as mixed

Chromosome	Start position	End position	Associated trait	Breed	Population frequency^a^	CNV type	Marginal R^2 b^	Flanking genes
8	15.37 Mb	15.38 Mb	Weight	HF	3	Deletion	0.0289	*bta-mir-873, bta-mir-876*
25	15.58 Mb	15.59 Mb	Weight	HF	7	Deletion	0.0337	*XYLT1*
16	16.55 Mb	16.56 Mb	Conformation	HF	17	Mixed	0.0002	*BRINP3*
20	43.06 Mb	43.08 Mb	Conformation	HF	4	Deletion	0.0314	*CDH6*
25	15.58 Mb	15.59 Mb	Conformation	HF	7	Deletion	0.0315	*XYLT1*
25	15.58 Mb	15.59 Mb	Fat	HF	7	Deletion	0.0296	*XYLT1*
10	24.59 Mb	24.60 Mb	Fat	LM	4	Mixed	0.0045	*TRAV16*

^a^The population frequency of the CNV is the number of animals in the population in which the CNV differed from the normal state

^b^The marginal R^2^ is the R^2^ which is attributable to the fixed effect, i.e. the CNV, in the linear mixed model

**Table 3:** Copy number variants (CNVs) associated with each of the carcass traits in Charolais (CH), Holstein-Friesian (HF), and Limousin (LM) with no obvious underlying pedigree relationship. When a CNV was present as both duplications and deletions in the population the CNV type was reported as mixed

Chromosome	Start position	End position	Associated trait	Breed	Population frequency^a^	CNV type	Marginal R^2 b^	Flanking genes
9	7.28 Mb	7.29 Mb	Weight	CH	3	Deletion	0.0201	*ADGRB3*
1	9.91 Mb	9.92 Mb	Weight	HF	6	Deletion	0.0276	*APP U6*
7	10.49 Mb	11.09 Mb	Weight	HF	3	Duplication	0.0286	*OR7A89, OR7A95*
22	61.33 Mb	61.38 Mb	Weight	HF	3	Mixed	0.0146	*CFAP100*
2	136.75 Mb	136.91 Mb	Conformation	HF	6	Mixed	0.0047	*U6, 5S rRNA*
7	23.81 Mb	23.86 Mb	Conformation	HF	25	Mixed	0.0053	*GRAMD2B*
11	103.48 Mb	103.50 Mb	Conformation	HF	3	Mixed	0.0044	*CAMSAP1*
11	49.90 Mb	49.91 Mb	Conformation	HF	3	Mixed	0.0056	*TMBSB10*
21	58.26 Mb	58.28 Mb	Conformation	HF	3	Mixed	0.0049	*ITPK1*
7	45.49 Mb	45.52 Mb	Conformation	LM	4	Deletion	0.0109	*APC2, PCSK4, REEP6*
10	39.73 Mb	39.73 Mb	Fat	LM	6	Deletion	0.0045	*RPL10L, U1*

^a^The population frequency is the number of animals in the population with a deletion or duplication variant of the CNV

^b^The marginal R^2^ is the R^2^ which is attributable to the fixed effect in the linear mixed model, in this case the CNV is the fixed effect

### Association analysis of the Log R-ratio value of single nucleotide polymorphisms

**Fig. 1:**
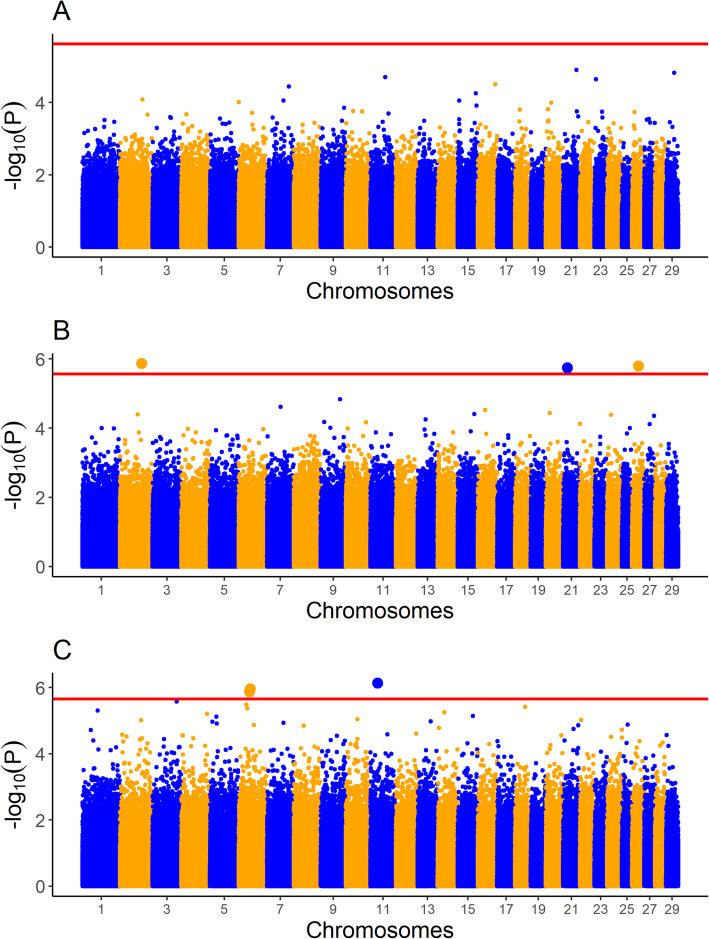
Manhattan plots for single nucleotide polymorphism (SNP) log R ratio (LRR) values associated with carcass weight in **A** Charolais **B** Holstein-Friesians, and **C** Limousins. The red line represents the significance threshold for each of the three breeds

**Fig. 2:**
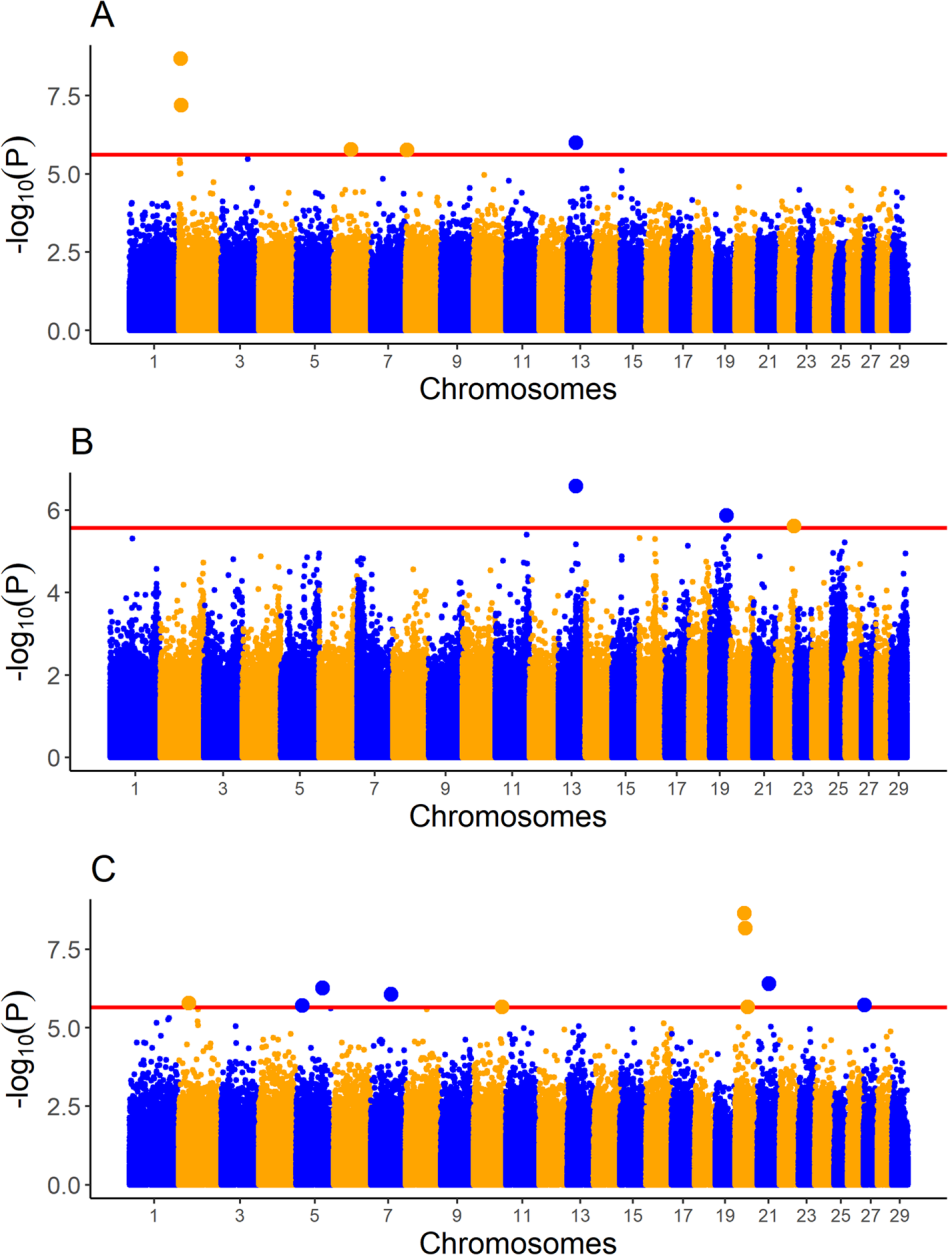
Manhattan plots for single nucleotide polymorphism (SNP) log R ratio (LRR) values associated with carcass fat in **A** Charolais **B** Holstein-Friesians, and **C** Limousins. The red line represents the significance threshold for each of the three breeds

**Fig. 3:**
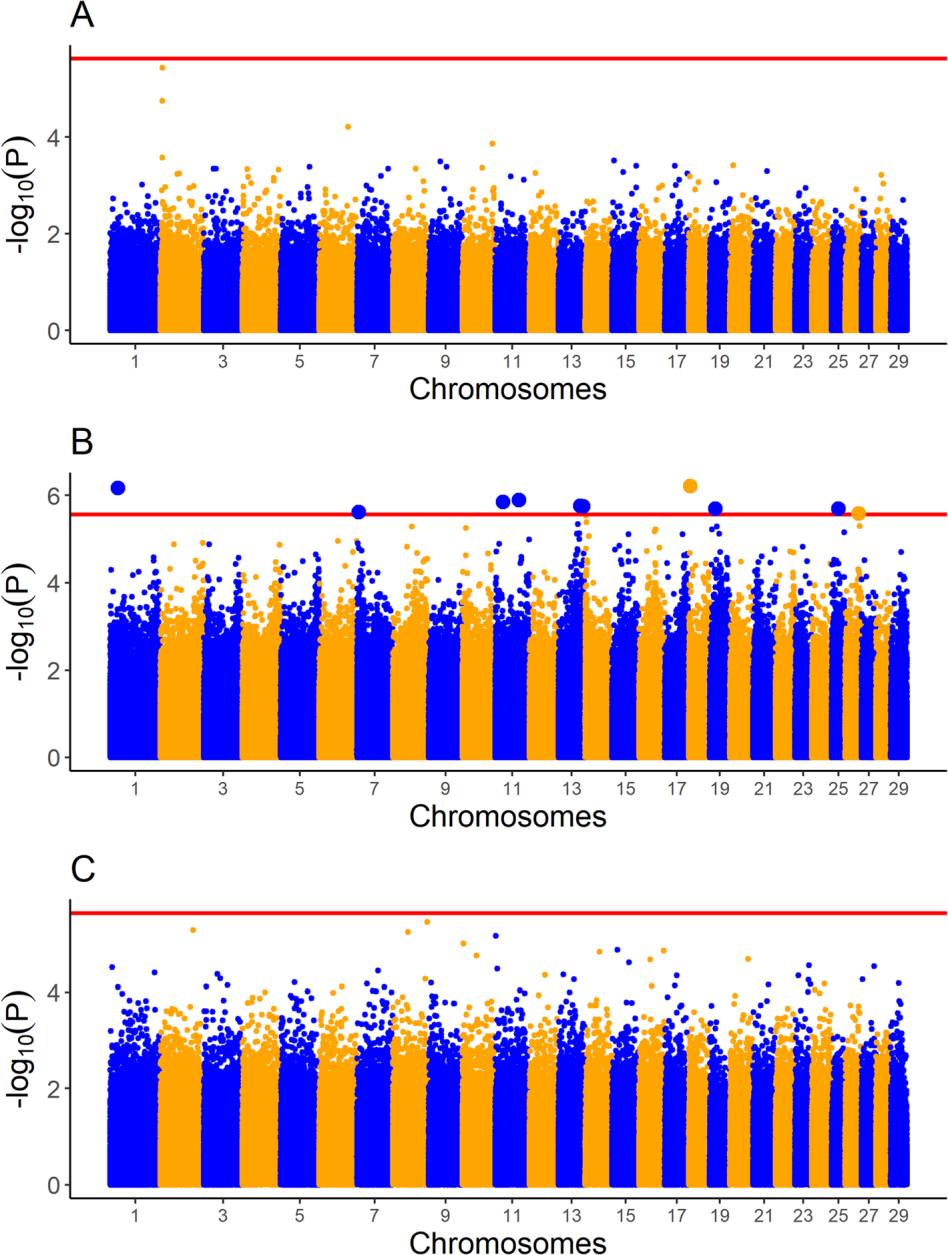
Manhattan plots of single nucleotide polymorphism (SNP) log R ratio (LRR) values associated with carcass conformation in **A** Charolais **B** Holstein-Friesians, and **C** Limousins. The red line represents the significance threshold for each of the three breeds

The LRR values of just 34 SNPs were associated with at least one carcass trait in at least one breed. None of the SNPs were associated with more than one trait, or were associated with the same trait in more than one breed. None of the associated SNPs overlapped in genomic position with any of the associated CNVs, or indeed were located within 500 kb upstream or downstream of the associated CNVs. The proportion of the variance attributable to each SNP LRR was between 0.0123 and 0.0287. A complete list of these SNPs and the R^2^ for each SNP is given in Table 4. Manhattan plots for the association between SNP LRR value and carcass weight, carcass fat, and carcass conformation in all three breeds are given in Figs. 1, 2 and 3, respectively.

**Table 4:** Single nucleotide polymorphisms (SNPs) with log R ratio (LRR) values associated with carcass weight, carcass fat, and carcass conformation in Charolais (CH), Holstein-Friesians (HF), and Limousins (LM)

Chromosome	Position	Breed	Associated trait	Marginal R^2 a^	Flanking genes
2	6.92 Mb	CH	Fat	0.0175	*MSTN*
2	8.01 Mb	CH	Fat	0.0136	*MSTN*
6	55.11 Mb	CH	Fat	0.0131	*ARAP2*
8	4.31 Mb	CH	Fat	0.0142	*GALNLT6*
13	27.45 Mb	CH	Fat	0.0240	*OPTN, MCM10*
2	90.47 Mb	HF	Weight	0.0287	*FZD7, CDK15*
21	18.53 Mb	HF	Weight	0.0194	*NTRK3*
26	27.13 Mb	HF	Weight	0.0287	*SORCS1*
1	24.98 Mb	HF	Conformation	0.0169	*ROBO2, 5S rRNA, U2*
7	5.59 Mb	HF	Conformation	0.0179	*PGLS, NIBAN3*
11	24.55 Mb	HF	Conformation	0.0233	*ELM4*
11	74.95 Mb	HF	Conformation	0.0176	*FAM228A, FAM228B*
13	68.22 Mb	HF	Conformation	0.0176	*FAM83D*
13	77.51 Mb	HF	Conformation	0.0157	*KCNB1*
18	4.24 Mb	HF	Conformation	0.0169	*MON1B*
19	17.08 Mb	HF	Conformation	0.0185	*ASIC2*
25	21.09 Mb	HF	Conformation	0.0155	*COG7*
26	42.62 Mb	HF	Conformation	0.0158	*SPADH1, U6*
13	55.30 Mb	HF	Fat	0.0175	*CDH4*
19	52.38 Mb	HF	Fat	0.0168	*SLC26A11*
22	58.14 Mb	HF	Fat	0.0146	*WNT7A, 5S rRNA*
6	44.26 Mb	LM	Weight	0.0177	*5S rRNA, U6, DHX15*
6	47.74 Mb	LM	Weight	0.0174	*5S rRNA*
11	30.81 Mb	LM	Weight	0.0167	*PPP1R21*
2	33.44 Mb	LM	Fat	0.0242	*7SK RNA*
5	20.16 Mb	LM	Fat	0.0124	*5S rRNA*
5	84.42 Mb	LM	Fat	0.0142	*LMNTD1*
7	64.07 Mb	LM	Fat	0.0131	*7SK RNA*
10	90.67 Mb	LM	Fat	0.0172	*NRXN3, 5S rRNA*
20	30.72 Mb	LM	Fat	0.0127	*U6*
20	33.02 Mb	LM	Fat	0.0143	*PLCXD3*
20	40.93 Mb	LM	Fat	0.0221	*NPR3*
21	37.67 Mb	LM	Fat	0.0123	*U6*
27	3.61 Mb	LM	Fat	0.0226	*CSMD1, U6*

^a^The marginal R^2^ is the proportion of the variance that was attributable to the fixed effect in a linear mixed model, which for this model is the SNP LRR

### Association analysis of single nucleotide polymorphism allele counts

To determine if a traditional association analysis using SNP allele counts could identify the same QTLs identified by the CNV analysis, the allele count of SNPs within 500 kb upstream or downstream of each associated CNV were tested for an association with the carcass trait in question. For each of the associated CNVs, there was between 121 and 356 SNPs located within the genomic region that spanned 500 kb upstream and downstream of the CNV. None of the SNP alleles located within 500 kb upstream or downstream of an associated CNV (including within the CNV itself) were associated with the carcass trait in question (P > 0.05). The same approach was used to determine if the called allele counts of any SNP located within 500 kb upstream or downstream of associated SNP LRRs (including the SNP itself) were also associated with the trait in question. For each of the associated SNP LRRs, there were between 164 and 486 SNPs located within the genomic region encompassing 500 kb upstream and downstream of the associated SNP LRR. The alleles of 30 SNPs located between 6.55 Mb and 8.43 Mb were associated with carcass fat in the Charolais; these 30 SNPs were located within 500 kb of two SNPs with LRRs also associated with carcass fat in the Charolais. The allele count of a single SNP located on chromosome 26 at 27.08 Mb was associated with carcass weight in Holstein-Friesians; this SNP was located 284.9 kb downstream of a SNP whose LRR value also associated with carcass weight in the Holstein-Friesians. There were 75 other SNPs located within the 284.9 kb region between the SNP associated by allele count and the SNP associated by LRR; none of these 75 SNPs associated with carcass weight in the Holstein-Friesians by either allele count or LRR. For all other detected SNP LRR associations, there was no SNP within 500 kb associated by allele count with the trait in question.

### Gene set enrichment analysis

A total of 72 genes overlapped in genomic position with the QTL regions of the 16 associated CNVs. The DAVID algorithm reported that there was no gene-set enrichment in this set of 72 genes. A total of 163 genes overlapped in genomic position with the QTL regions of the associated SNP LRRs, and there was no gene set enrichment in these 163 genes.

## Discussion

Carcass weight, carcass conformation, and carcass fat are key components of carcass value and have a direct impact on the profitability of beef farming [2]. Considerable emphasis is placed on carcass traits in beef-on-beef breeding goals [25, 26], beef-on-dairy breeding goals [27], and even dairy-on-dairy breeding goals [28]. Given the economic importance of carcass traits in cattle, an understanding of the genetic variants that contribute to the underlying inter-animal variability is of practical importance for any breeding or management strategy in cattle.

While several previous studies have attempted to relate SNP allele count data to carcass traits [7, 8, 29], the present study is one of the few studies to perform genome-wide association analyses between CNVs and carcass traits in cattle, and to the best of our knowledge is the only study to use a multi-breed population of cattle. Furthermore, to the best of our knowledge this is also the first genome-wide association analyses of carcass traits in cattle using SNP LRR data. While 2,842 animals were included in the analyses, these animals were sires, and their phenotypes were breeding values estimated from their descendants; in fact, the 2,842 animals were the equivalent of > 150,000 effective own phenotypic records.

In the chromosome-based association analyses, four different analyses were conducted. Within each of these analyses, the 29 autosomal chromosomes were separately tested for an association with each of the three carcass traits in the three different breeds; this amounts to 1,044 individual tests. Given that an association was detected for only two of these tests (i.e., the proportion of chromosome 20 with a called duplication CNV and carcass conformation in the Holstein-Friesians; the mean LRR of chromosome 13 and carcass conformation in Holstein-Friesians), it suggests that these results may not reflect true associations but rather were spurious associations due to noise in the data.

### Shared quantitative trait loci

A single CNV located on chromosome 25 at 15.58 Mb was associated with all three carcass traits but just in Holstein-Friesians (Table 2). This CNV was one of the 5 CNVs (Table 2) for which the majority of the animals with the CNVs were half-siblings, but per a power analysis there was an insufficient number of animals to test for a difference in the mean deregressed EBVs between half-siblings with the CNV versus those without the CNV. The association of this CNV with each of the carcass traits may have been due to underlying population stratification rather than a true association between the CNV and each of the carcass traits in Holstein-Friesians.

Although no SNP LRRs were associated with more than one trait, the SNP LRR with the strongest association with carcass fat in Charolais, located on chromosome 2 at 6.85 Mb, was also the SNP LRR with the strongest association with carcass conformation in the Charolais. However, this association with carcass conformation (*P* = 3.715 × 10^− 6^ prior to multiple testing adjustment) was not above the threshold for significance after adjusting for multiple testing albeit there was a strong association. The lack of QTLs shared across the different breeds or traits for either the associated CNVs or the associated SNP LRRs is broadly consistent with the literature using SNP allele count in the association analysis of carcass traits in cattle, where the majority of associated SNPs are only associated with one particular carcass trait within a single breed [7, 30]. The CNVs available for testing were not all unique to one breed; many of the CNVs were shared between the Holstein-Friesian, Charolais, and Limousin populations in the present study. Therefore, it was possible for a CNV to be associated with a given trait in more than one breed. For each of the 16 CNVs associated with at least one trait in at least one breed, the P-value for association with each of the other two traits in the other breeds is given in supplemental Table S1. Small sample bias may be contributing to the absence of shared QTLs between the different breeds and traits. Large populations are required to detect associations of small effect [31], so it is possible that QTLs associated with one trait in one breed are also associated with that trait in another breed or trait but the effect was too small to be detected in the present study.

None of the associated SNP LRRs overlapped in genomic position with any of the tested CNVs in the present study. The fact that there were no CNVs detected in the genomic regions of the associated SNP LRRs may be due to the reported high false negative rate of CNV detection [32, 33]. Copy number variants in the present study with fewer than 3 SNPs cannot be detected using the CNV-calling algorithm PennCNV, but it is likely, based on the population frequency of CNVs by genomic length, that many CNVs, or indeed InDels, do exist below this minimum length threshold for detection [34].

### Comparison with other genome-wide association studies

Zhou et al. [18] performed a genome-wide CNV association analysis of carcass traits in a population of 2,230 Nelore cattle and discovered 17 CNVs that associated with carcass traits or growth traits. There was, however, no overlap in the genomic position of the associated CNVs identified in the present study with those documented by Zhou et al. [18]. This is not surprising given that both studies used different breeds and associated genetic variants mostly tend to be breed specific in genome-wide association analyses [7, 29, 30].

No SNP genotypes located within 500 kb of an associated CNV in the present study associated with the trait of interest in the given breed; this, therefore, suggests that the CNVs and SNP genotypes in the present study had independent contributions to the carcass traits analysed. Nonetheless, the QTL regions of associated CNVs and associated SNP LRRs were compared to QTL regions reported in other genome-wide SNP genotype association analyses of carcass traits [7], muscularity traits [29], and skeletal traits [35]; each of these studies used imputed sequence data and similar cattle populations to the population used in the present study. The studies on muscularity and skeletal traits were also included in this comparison because muscularity and skeletal traits are strongly correlated with carcass traits in cattle [36, 37]. A CNV associated with carcass conformation in the Holstein-Friesians, located on chromosome 7 between 28.81 Mb and 28.86 Mb, overlapped in genomic position with a QTL associated with stature in Holstein-Friesians [35]. None of the other associated CNVs overlapped in genomic position with QTLs reported by either Doyle et al. [29], Doyle et al. [35], or Purfield et al. [7]. Two SNP LRRs associated with carcass fat in the Charolais, which were located on chromosome 2 at 6.85 Mb and 8.01 Mb, overlapped in genomic position with QTLs documented by Purfield et al. [7], which were associated with carcass weight, carcass fat, and carcass conformation in Charolais. The same two SNPs overlapped with QTLs associated with wither width, thigh width, inner thigh, hind quarter [29], as well as back length [35] in Charolais. This genomic region was also identified in the present study using the SNP allele count association analysis. It is likely that the association of these two SNP LRRs with carcass fat is due to their close proximity to the *MSTN* gene. The *MSTN* gene encodes the protein Myostatin whose function is to regulate muscle cell proliferation [38]. Mutations in *MSTN* are known to have large effect on carcass traits in cattle [38–40].

In addition to comparisons with other genome-wide association studies, the QTL regions of the associated CNVs were compared to the Ensembl genome browser to determine if these genomic regions had previously been annotated with CNVs. For 14 of the 16 associated CNV QTLs, the genomic region of the detected CNV QTL in the present study had previously been annotated with CNVs as per the Ensembl genome browser. The QTL of the CNV located on chromosome 2 between 136.6 Mb and 137.0 Mb did not overlap with a CNV region. Similarly, the QTL of the CNV located on chromosome 22 between 61.2 Mb and 61.5 Mb did not overlap with a reported CNV region as per the Ensembl genome browser.

### Candidate genes

In the present study two SNP LRRs upstream of the *MSTN* gene were associated with carcass fat in the Charolais; furthermore SNP allele counts in this region were also associated with carcass fat in the Charolais. Given that the genomic region flanking the *MSTN* gene was detected in this study using both SNP LRR and SNP allele counts, it validates that SNP LRR data can be used to detect associations between carcass traits and the genes that influence carcass traits in cattle. Other genes known to have a large effect on carcass traits in cattle such as *LCORL* [41–43], *NCAPG* [44, 45], and *PLAG1* [42, 46] were not identified in the present study. For all three breeds, there were CNVs and SNPs at or near the genomic locations of *LCORL*, *NCAPG*, and *PLAG1*; therefore, it was possible for the association analyses to detect these genomic regions. It could be the case that the SNP LRRs and CNVs flanking these large effect genes are not in linkage disequilibrium with the causative mutations in these genes, nor are these genes directly influenced by copy number variation in the present study.

In addition to *MSTN*, there are several other plausible candidate genes which may be associated with carcass traits in cattle. The *APP* gene, which encodes the amyloid precursor protein, flanks a CNV located on chromosome 1 at 9.91 Mb which was associated with carcass weight in the Holstein-Friesians. The *APP* gene is associated with meat tenderness in Hanwoo cattle [47] and *APP* has also been identified as a candidate gene associated with hip width in Holstein-Friesians [35]. A CNV located on chromosome 10 at 39.73 Mb associated with carcass fat in the Limousins is located close to *RPL10L*, a gene which encodes ribosomal protein L10. The expression of *RPL10L* is associated with residual feed intake in Hereford Angus crossbreed steers [48]. In the present study, one CNV was associated with all three carcass traits in the Holstein-Friesians; this CNV located on chromosome 25 at 15.58 Mb overlaps in genomic position with the *XYLT1* gene. The *XYTL1* gene is associated with growth traits in Canchim cattle [49], and was also reported as a candidate gene associated with hip width in Angus cattle [35].

The genes near or nearest to an associated CNV or SNP LRRs were often components of the spliceosome or the ribosome (Tables 2, 3 and 4). In metazoan animals, cattle included, multiple copies of the genes encoding the subunits of the spliceosome and ribosome are maintained in the genome [50, 51]. Several distinct copies of *U6* were located near CNVs associated with carcass conformation and carcass weight in the Holstein-Friesians (Table 3). Likewise, several distinct copies of *U6* were located in close proximity to SNPs with LRRs associated with carcass conformation and carcass fat in Holstein-Friesians, carcass fat in Charolais, and carcass weight and carcass fat in Limousins (Table 4). Multiple distinct copies of the *5S rRNA* gene, which encodes an RNA (rRNA) subunit of the ribosome [52], were located in close genomic proximity to associated CNVs and associated SNP LRRs (Tables 3 and 4).

The close genomic proximity of several distinct copies of *U6* and *5S rRNA* genes with associated CNVs and SNP LRRs indicates that these genes may be associated with carcass traits in cattle. However, it could be the case that these genes were often located near associated CNVs and SNP LRRs because they are enriched in genomic regions frequently subject to copy number variation, due to the fact that there are multiple copies of these genes in the cattle genome. Other cattle studies have also reported that *U6* and *5S rRNA* are under positive selection, and are also associated with carcass traits. Both *U6* and *5S rRNA* genes have been demonstrated to be under positive selection in North African cattle [53], as well as in Holstein, Hanwoo, and N’Dama cattle [54]. Zhang et al. [55] reported that *5S rRNA* and *U6* may be candidate genes for dry matter intake in a multi-breed population of cattle. Additionally, Wang et al. [8] reported that *U6* may be a candidate gene for average back fat thickness and carcass marbling, and *5S rRNA* may be a candidate gene for carcass marbling in a multi-breed population of cattle. In addition to *5S rRNA*, other genes related to the structure and function of the ribosome have also been associated with carcass traits in cattle. The *RPS20* gene, which encodes a protein that is part of the structure of the ribosome [56], is associated with animal stature in Hanwoo cattle [57]. Mutations in *L27a*, a ribosomal subunit, are associated with marbling in Japanese cattle [58]. Similar to the ribosome, the spliceosome may have an impact on carcass traits in cattle. A transcriptomics study in cattle reported that alternatively spliced mRNA transcripts are associated with intramuscular fat content and cross-sectional area of muscle in Nelore cattle [59].

## Conclusions

Genomic regions associated with carcass traits in cattle were identified using both CNV and SNP LRR data, which, for the vast majority, would not have been detected using traditional genome wide association approaches based on SNP allele counts. Hence SNP allele counts, SNP LRR data, and CNV data could be complementary in detecting genomic regions associated with performance. Although only a small proportion of the genetic variability in the carcass traits was captured with these two new variant types, the same may not be true for other populations or traits; moreover, improvements in the calling of CNVs in particular could possibly improve the strength of the association analyses.

## Materials and methods

### Genotype data

All animals were genotyped using the Illumina BovineHD SNP array (777,962 SNPs) (Illumina Inc., San Diego, CA); the positions of all SNPs were taken from the UMD 3.1 build of the bovine genome [60]. Only animals with at least 95 % of their SNPs called were considered. Individual SNPs with a call rate of less than 95 % were excluded, as were SNPs on the X or Y chromosomes, and SNPs without a reported chromosome or position. In addition, 1,611 SNPs inconsistent with Mendelian inheritance in more than 2 % of parent-progeny pairs [61] were also excluded from the analyses. After edits 712,555 SNPs were available for 1,324 Holstein-Friesian, 981 Charolais, and 1,129 Limousin bulls.

### Phenotypic data

Estimated breeding values (EBVs) and associated reliability estimates for carcass weight, carcass fat, and carcass conformation were obtained for each animal from the January 2019 national genetic evaluation of the Irish Cattle Breeding Federation (ICBF) (Bandon, Co. Cork, Ireland). In Ireland, carcass weight is the weight of the carcass after the head, limbs, hide, internal organs, and visceral fat are removed from the carcass [4]. Carcass conformation and carcass fat are both scored on a 15-point scale according to the EU beef carcass classification system (EUROP) using video image analysis [62]. The Secant method described by Stranden and Mantysaari [63] was used to deregress the EBVs of each trait using the Mix99 software [64]. The effective record contribution (ERC) was calculated for each animal using the method described by Harris and Johnson [65]. Animals with an ERC < 1 were excluded from further analysis. The number of animals available after edits and the sum of the ERCs for each breed and trait is presented in supplemental Table S2. Summary statistics on the deregressed EBVs for each breed are given in supplemental Table S3.

### Detection of copy number variants

During the genotyping process, when a DNA molecule complementary to a particular SNP binds to the SNP array it precipitates a fluorescence reaction; the intensity of this reaction is recorded in the log R-ratio (LRR) statistic [21]. The LRR value is the observed fluorescence intensity for the SNP divided by the expected fluorescence of the SNP, where the expected fluorescence intensity of the SNP is derived by linear interpolation between the called genotype clusters [22]. A related statistic to the LRR value is the B allele frequency (BAF), the BAF of a SNP is the fluorescence intensity of the B allele at the SNP divided by the total fluorescence intensity of the SNP. PennCNV [66] and QuantiSNP [67] are two freely available CNV-calling algorithms that use the LRR and BAF values of SNPs to detect CNVs using a hidden Markov model approach. In the present study, both algorithms were used to call CNVs separately from each animal in the population; two algorithms were used to call CNVs separately from each animal in the population, as has been previously suggested by Winchester et al. [68], because both algorithms are reported to have low false positive rates of CNV discovery [32, 66, 67], but have higher false negative rates of CNV discovery. The guanine-cytosine (GC) content of DNA is known to bias CNV detection using SNP array data [69]. To account for the GC content of DNA, an adjustment was made to account for the correlation between the LRR values of SNPs and the GC content of DNA flanking 500 kb upstream and downstream of the SNP. PennCNV specifies that each CNV must contain at least 3 SNPs so for consistency between both algorithms, the same criterion was applied to the CNVs called by QuantiSNP; no maximum length for a CNV was specified in the present study.

A CNV was considered to have been called by both PennCNV and QuantiSNP when the endpoints of the CNV called by one algorithm, were no more than 1 SNP from the endpoints of the CNV called by the other algorithm. This difference of 1 SNP was allowed for because if there is an error in endpoint demarcation, the true endpoint of the CNV is typically only one SNP away [32, 67]. Copy number variants called by either PennCNV or QuantiSNP were included in the CNV dataset, but CNVs called by both PennCNV and QuantiSNP were not double counted. The final CNV data-set consisted of CNVs called by either PennCNV or QuantiSNP whose copy number deviated from the normal state (i.e. two copies) in more than 3 animals within a given breed. Summary statistics on the CNVs available after edits in the present study are given in supplementary Table S4.

### Population structure

The population structure for each breed was inferred using the method described by Patterson et al. [70] using the imputed SNP genotype data (712,555 SNPs) for each animal within breed. In this method, eigen decomposition is performed on a covariance matrix that is equivalent to the genomic relationship matrix calculated using method 1 of VanRaden [71]. Eigen decomposition was carried out separately on the genomic relationship matrices for the Charolais, Holstein-Friesians, and Limousins. Large eigenvalues, relative to a population without population structure, is indicative of population structure [70].

### Association analyses

The present study consisted of four separate sets of association analyses of carcass weight, carcass fat, and carcass conformation; two were based on aggregate metrics per chromosome with the remaining two being undertaken for individual CNV or SNP. The four separate analyses differed by the independent variable in the model which were: (1) the mean LRR per autosomal chromosome, (2) the proportion of each chromosome that had a called CNV, (3) the CNV copy number, and (4) the SNP LRR value. The proportion of the chromosome that had a called CNV was calculated per animal as the combined genomic length of all CNVs on the chromosome divided by the genomic length of the chromosome. This calculation was also repeated separately using only called deletion CNVs to obtain the proportion of the chromosome with a called deletion CNV; similarly, the proportion of the chromosome with a called duplication CNV was calculated separately using only called duplication CNVs. The genomic length of the chromosome was calculated as the genomic distance between the first and last genotyped SNP on the chromosome.

The motivation for the CNV copy number association analysis was to determine if individual CNVs were associated with any of the three carcass traits considered. Similarly, the purpose of the SNP LRR association analysis was to determine if individual SNP LRR data can be used to identify genomic regions associated with carcass traits in cattle. Furthermore, it was of interest to determine if common genomic regions existed between those identified by the CNV copy number and those with SNP LRR, since SNP LRR is an important statistic for detecting CNVs from SNP array data. There may be an association between CNV burden and traits of interest; for example, previous studies in humans have reported associations between CNV burden and both autism spectrum disorders and schizophrenia [72, 73]. The association analysis of the proportion of each chromosome that has a called CNV was to determine if CNV burden per chromosome was associated with any of the carcass traits in cattle. Similarly the association analysis of mean LRR per chromosome was carried out since mean LRR per chromosome may also be reflective of CNV burden.

Each association analysis was undertaken separately for each of the three breeds and each of the three carcass traits using the R package lme4qtl [74]. A linear mixed model approach was used to model the association between the independent variable and the deregressed EBV values:

$$\mathbf{dEBV}\boldsymbol\;\boldsymbol=\boldsymbol\;\mathbf\mu\boldsymbol\;\boldsymbol+\boldsymbol\;\mathbf{Xgi}\boldsymbol\;\boldsymbol+\boldsymbol\;\mathbf{Zu}\boldsymbol\;\boldsymbol+\boldsymbol\;\mathbf e$$

where **dEBV** was the vector of deregressed EBVs, **µ** was a vector representing the intercept term, and **g**_**i**_ was a vector of the i^th^ independent variable in the model. In the mean LRR value per chromosome analyses, **g**_**i**_ was a vector of mean LRR value per animal for the i^th^ chromosome treated as a continuous fixed effect while in the analyses of the proportion of the chromosomes with a called CNV, **g**_**i**_ was a vector of the proportion of the chromosome with a called CNV for the i^th^ chromosome treated as a continuous fixed effect. In the CNV association analyses, **g**_**i**_ was a vector of the copy number for the i^th^ CNV treated as a categorical fixed effect, while in the SNP LRR association analyses, **g**_**i**_ represented a vector of LRR values for the i^th^ SNP treated as a continuous fixed effect. In all models, **u** represented the polygenic effect of each animal in the population treated as a random effect and **e** represents a vector of random residual effects. In all models, **X** and **Z** were design matrices that related the fixed and random effects to each animal record. In all models, population stratification was accounted for by fitting a direct additive genetic effect via a genomic relationship matrix. Method 1 of VanRaden [71] was used to generate the genomic relationship matrix separately for each breed from the edited set of autosomal SNPs (n = 712,555); missing genotypes in the edited set of SNPs were imputed using the imputation software suite FImpute [75]. The random effect, **u**, was assumed to have the distribution N(0, **G**${{\upsigma }}_{a}^{2}$) where **G** represents the genomic relationship matrix and ${{\upsigma }}_{a}^{2}$ represents the additive genetic variance. The random residual error term, **e**, was assumed to have the distribution N(0, **I**${{\upsigma }}_{e}^{2}$), where **I** is the identity matrix and ${{\upsigma }}_{e}^{2}$ is the residual variance. The deregressed EBVs were weighted to account for differences in the reliabilities of the deregressed EBVs within the population. The weighting on each record was calculated as detailed in Garrick et al. [76],

$$\mathrm w=(1-h^2)/\left[c+\frac{1-r_i^2}{r_i^2}\right]h^2$$

where h² is the heritability of the trait, r_i_^2^ is the reliability of the EBV for i^th^ animal, and c is the proportion of genetic variation not accounted for by the genetic variant in the model. The value of c was set to 0.9 for each trait [77].

PennCNV and QuantiSNP both report four different copy number states; these are double copy deletion, single copy deletion, single copy duplication, and double copy duplication. In the CNV association analysis, the CNV copy number classes double-deletion, single-deletion, no CNV, single-duplication, and double-duplication, were recoded as 0, 1, 2, 3, and 4, respectively. For any given CNV, if there was less than 5 animals in the population with the double-deletion variant of the CNV, or less than 5 animals with the single-deletion variant, both deletion classes were collapsed together. Similarly, if there was less than 5 animals in the population with the double-duplication variant, or less than 5 animals with the single-duplication variant, both duplication classes were collapsed together. All of the CNVs available in the present study had at most 3 classes because there was no CNV that had both 5 single-deletion and 5 double-duplication variants of the CNV, or 5 single-duplication and 5 double-duplication variants of the CNV. The number of CNVs available for each breed and trait, after edits, is given in Table 1.

### Model testing

For the analyses at the chromosomal level, the P-values related to the estimate of the regression coefficient in each model was adjusted by multiplying the P-value by the number of chromosomes tested to account for multiple testing. When CNV copy number and SNP LRR were the genomic features of interest, the number of independent tests was calculated separately for each dataset as the number of principal components required to account for 99.5 % of the variation in the data [78, 79]. The P-value relating to the estimate of the regression coefficient in each model was adjusted by multiplying the P-value by the number of independent genomic regions. Genomic variants with an adjusted P-value ≥ 0.05 were not considered further.

The proportion of the variance attributable to associated CNVs and SNP LRRs was calculated separately for each variant using the marginal R^2^ statistic as described by Nakagawa and Schielzeth [80]. The marginal R^2^ statistic calculates the proportion of the variance attributable to the fixed effect in a linear mixed model.

### Gene set enrichment analysis

For gene-set enrichment analysis, a QTL region flanking each of the associated CNVs and SNP LRRs was specified as the genomic region spanning 500 kb upstream and 500 kb downstream of the associated CNV or SNP. The genomic position of each associated SNP LRR was updated per the ARS_UCD1.2 assembly of the bovine genome [81]. For each breed and trait analysed, the sets of genes which overlapped with the associated QTL regions were obtained using Ensembl Biomart (http://ensembl.org) based on the ARS_UCD1.2 build of the bovine genome [81]. The list of genes that overlapped with the QTL regions of the associated genetic variants were evaluated for gene set enrichment using the Database for Annotation, Visualization, and Integrated Discovery (DAVID) version 6.8 [82]. The DAVID algorithm clusters the genes by function and gives an enrichment score and P-value to each cluster under the null hypothesis that there was no gene-set enrichment.

## Supplementary Information

**Additional file 1:Table S1.** The *P*-value for the association between each of the associated CNVs with each of the three traits across all three breeds. The genomic location column is formatted chromosome, start position bp, end position bp. Where a value of NA is given, the CNV was not tested for the given breed and trait due to insufficient population frequency of the CNV in that population.

**Additional file 2: Table S2.** The number of animals available for each breed and trait, the mean effective record contribution (ERC) per animal, and the sum of the ERCs for all animals available for each breed and trait.

**Additional file 3: Figure S1.** Manhattan plots for copy number variants (CNVs) associated with carcass weight in **A**) Charolais **B**) Holstein-Friesians **C**) Limousins. The red line represents the significance threshold for each of the three breeds.

**Additional file 4: Figure S2.** Manhattan plots for copy number variants (CNVs) associated with carcass fat in **A**) Charolais **B**) Holstein-Friesians **C**) Limousins. The red line represents the significance threshold for each of the three breeds.

**Additional file 5: Figure S3.** Manhattan plots for copy number variants (CNVs) associated with carcass conformation in **A**) Charolais **B**) Holstein-Friesians **C**) Limousins. The red line represents the significance threshold for each of the three breeds.

**Additional file 6: Table S3.** The mean, standard deviation, minimum, and maximum of the deregressed estimated breeding values (EBVs) for each trait for each of the three breeds.

**Additional file 7: Table S4.** Median, mean, and standard deviation of the number of CNVs per animal, referred to as count, and the length of CNVs within breed.

## Data Availability

The genotype and phenotype datasets analysed in the present study are available from the corresponding author upon reasonable request.

## Abbreviations

BAF: B allele frequency
CH: Charolais
CNV: Copy number variant
DAVID: Database for annotation, visualisation, and integrated discovery
EBV: Estimated breeding value
ERC: Effective record contribution
GC: Guanine cytosine
HF: Holstein-Friesian
ICBF: Irish Cattle Breeding Federation
InDel: Insertion deletion
Kb: Kilobase pair
LM: Limousin
LRR: Log R-ratio
Mb: Megabase pair
SNP: Single nucleotide polymorphism
