# Supplementary material for: Genome-wide association analyses of carcass traits using copy number variants and raw intensity values of single nucleotide polymorphisms in cattle
Source: BMC Genomics. 2021 Oct 23;22:757. doi: 10.1186/s12864-021-08075-2 (PMC8542340; doi:10.1186/s12864-021-08075-2)
Supplement: Supplementary file 1 — Additional file 1:Table S1. The P-value for the association between each of the associated CNVs with each of the three traits across all three breeds. The genomic location column is formatted chromosome, start position bp, end position bp. Where a value of NA is given, the CNV was not tested for the given breed and trait due to insufficient population frequency of the CNV in that population. [file 12864_2021_8075_MOESM1_ESM.docx]

Table S1.The P-value for the association between each of the associated CNVs with each of the three traits across all three breeds. The genomic location column is the format chromosome, start position bp, end position bp. Where a value of NA is given, the CNV was not tested for the given breed and trait due to insufficient population frequency of the CNV in that population.

| Genomic location | Holstein-Friesian | | | Charolais | | | Limousin | | |
| --- | --- | --- | --- | --- | --- | --- | --- | --- | --- |
|  | Weight | Conformation | Fat | Weight | Conformation | Fat | Weight | Conformation | Fat |
| 10:24587769-24604106 | 0.125 | 0.999 | 0.601 | NA | NA | NA | 0.638 | 2.034x10^-11^ | 0.364 |
| 10:39727908-39734995 | 0.718 | 0.713 | 0.920 | NA | NA | NA | 0.270 | 1.803 x10^-11^ | 0.016 |
| 11:103484884-103495904 | NA | 7.281 x10^-9^ | 5.422 x10^-4^ | NA | NA | NA | NA | NA | NA |
| 11:49897668-49912381 | NA | 7.245 x10^-9^ | 5.426 x10^-4^ | NA | NA | NA | NA | NA | NA |
| 16:65553784-65561570 | 0.101 | 5.551 x10^-16^ | 0.003 | 0.696 | 0.135 | 0.165 | 0.108 | 0.458 | 0.031 |
| 1:9914773-9917417 | 2.215 x10^-7^ | 0.721 | 0.041 | NA | NA | NA | NA | NA | NA |
| 20:43055922-43081692 | 2.411 x10^-5^ | 4.892 x10^-7^ | 1.866 x10^-4^ | 0.440 | 0.728 | 0.888 | NA | NA | NA |
| 2:136749793-136908437 | 0.673 | 2.478 x10^-6^ | 0.003 | NA | NA | NA | 0.190 | 0.717 | 0.655 |
| 21:58257863-58277611 | NA | 7.257 x10^-9^ | 5.425 x10^-4^ | NA | NA | NA | NA | NA | NA |
| 22:61332462-61379134 | 4.495 x10^-7^ | 0.002 | 0.003 | NA | NA | NA | NA | NA | NA |
| 25:15577684-15585727 | 1.271 x10^-6^ | 5.262 x10^-7^ | 3.428 x10^-7^ | 0.827 | 0.231 | 0.351 | 0.471 | 0.619 | 0.168 |
| 7:10486424-11091861 | 3.196 x10^-7^ | 0.636 | 0.837 | NA | NA | NA | NA | NA | NA |
| 7:23813839-23855608 | 0.973 | 4.571 | 0.060 | 0.510 | 0.428 | 0.251 | 0.806 | 0.120 | 0.816 |
| 7:45487894-45519837 | 0.240 | 0.574 | 0.629 | NA | NA | NA | 0.040 | 1.666 x10^-5^ | 0.008 |
| 8:15374573-15387156 | 5.221 x10^-7^ | 0.722 | 0.061 | 0.890 | 0.817 | 0.229 | NA | NA | NA |
| 9:7275154-7285017 | NA | NA | NA | 7.745 x10^-7^ | 0.184 | 0.005 | NA | NA | NA |
