# Supplementary material for: Genome-wide association analyses of carcass traits using copy number variants and raw intensity values of single nucleotide polymorphisms in cattle
Source: BMC Genomics. 2021 Oct 23;22:757. doi: 10.1186/s12864-021-08075-2 (PMC8542340; doi:10.1186/s12864-021-08075-2)
Supplement: Supplementary file 2 — Additional file 2: Table S2. The number of animals available for each breed and trait, the mean effective record contribution (ERC) per animal, and the sum of the ERCs for all animals available for each breed and trait. [file 12864_2021_8075_MOESM2_ESM.docx]

Table S2. The number of animals available for each breed and trait, the mean effective record contribution (ERC) per animal, and the sum of the ERCs for all animals available for each breed and trait.

| Breed | Trait | Number of animals | Total ERC | Mean ERC per animal |
| --- | --- | --- | --- | --- |
| Charolais | Weight | 945 | 26,070 | 27.6 |
|  | Conformation | 945 | 32,478 | 34.4 |
|  | Fat | 945 | 37,326 | 39.5 |
| Holstein-Friesian | Weight | 892 | 49,699 | 55.7 |
|  | Conformation | 915 | 61,942 | 67.7 |
|  | Fat | 923 | 71,197 | 77.1 |
| Limousin | Weight | 974 | 29,917 | 30.7 |
|  | Conformation | 973 | 42,841 | 44.0 |
|  | Fat | 974 | 37,274 | 38.3 |
