# Supplementary material for: Genome-wide association analyses of carcass traits using copy number variants and raw intensity values of single nucleotide polymorphisms in cattle
Source: BMC Genomics. 2021 Oct 23;22:757. doi: 10.1186/s12864-021-08075-2 (PMC8542340; doi:10.1186/s12864-021-08075-2)
Supplement: Supplementary file 4 — Additional file 4: Figure S2. Manhattan plots for copy number variants (CNVs) associated with carcass fat in A) Charolais B) Holstein-Friesians C) Limousins. The red line represents the significance threshold for each of the three breeds. [file 12864_2021_8075_MOESM4_ESM.docx]

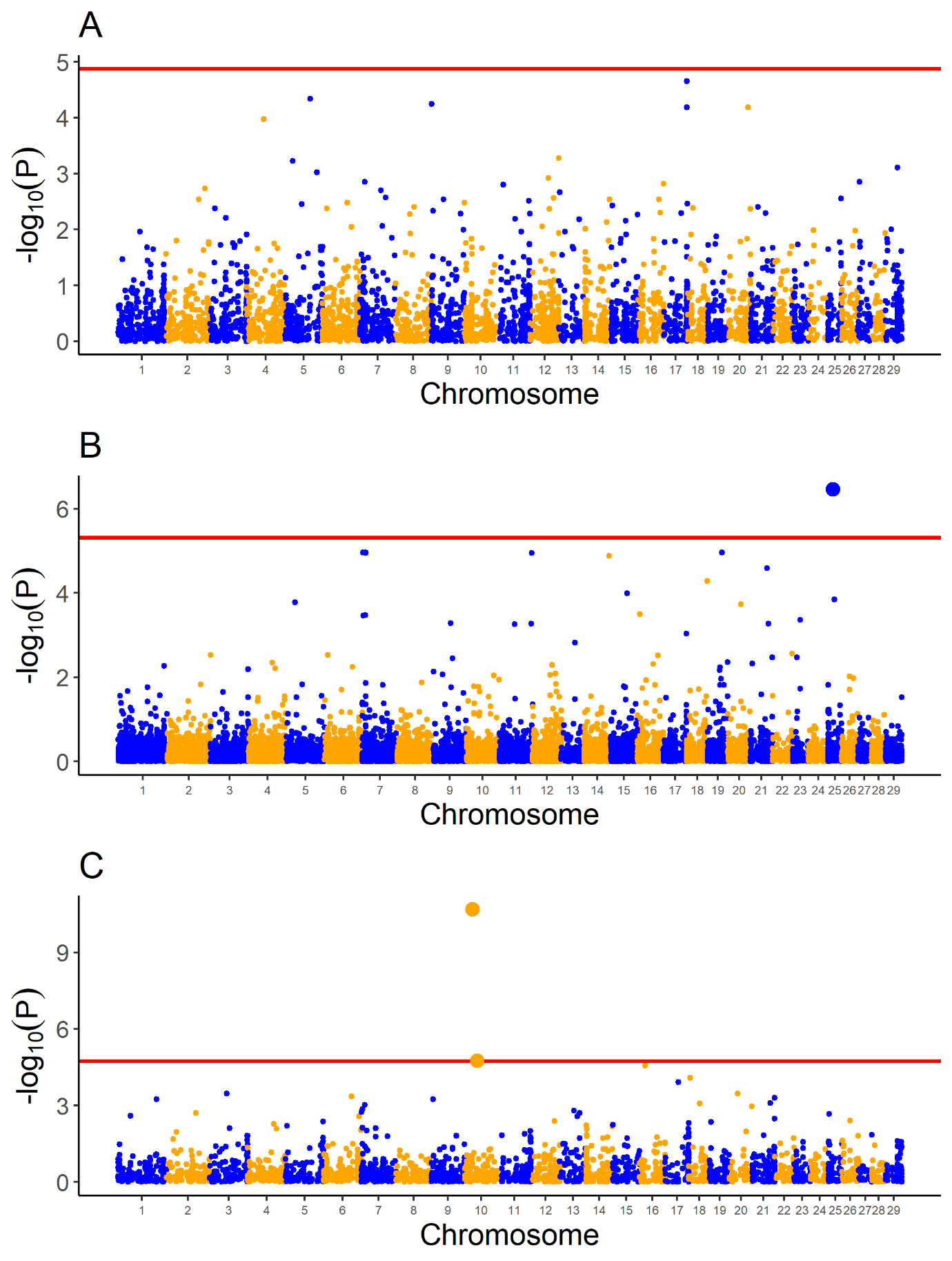
 Figure S2. Manhattan plots for copy number variants (CNVs) associated with carcass fat in **A**) Charolais **B**) Holstein-Friesians **C**) Limousins. The red line represents the significance threshold for each of the three breeds.
