# Supplementary material for: Genome-wide association analyses of carcass traits using copy number variants and raw intensity values of single nucleotide polymorphisms in cattle
Source: BMC Genomics. 2021 Oct 23;22:757. doi: 10.1186/s12864-021-08075-2 (PMC8542340; doi:10.1186/s12864-021-08075-2)
Supplement: Supplementary file 6 — Additional file 6: Table S3. The mean, standard deviation, minimum, and maximum of the deregressed estimated breeding values (EBVs) for each trait for each of the three breeds. [file 12864_2021_8075_MOESM6_ESM.docx]

Table S3: The mean, standard deviation, minimum, and maximum of the deregressed estimated breeding values (EBVs) for each trait for each of the three breeds.

| Breed | Carcass trait | Mean | Standard Deviation | Minimum | Maximum |
| --- | --- | --- | --- | --- | --- |
| Charolais | Weight | 28.06 | 13.74 | -48.37 | 99.86 |
| Charolais | Conformation | -0.28 | 0.42 | -2.13 | 1.70 |
| Charolais | Fat | 1.76 | 0.45 | -0.65 | 4.76 |
| Holstein-Friesian | Weight | -3.363 | 12.09 | -79.20 | 64.27 |
| Holstein-Friesian | Conformation | -0.23 | 0.58 | -5.61 | 3.60 |
| Holstein-Friesian | Fat | -0.58 | 0.68 | -5.18 | 4.89 |
| Limousin | Weight | 18.55 | 15.55 | -93.07 | 148.96 |
| Limousin | Conformation | -0.21 | 0.46 | -4.27 | 1.45 |
| Limousin | Fat | 1.90 | 0.58 | -1.73 | 9.61 |
