# Supplementary material for: Genome-wide association analyses of carcass traits using copy number variants and raw intensity values of single nucleotide polymorphisms in cattle
Source: BMC Genomics. 2021 Oct 23;22:757. doi: 10.1186/s12864-021-08075-2 (PMC8542340; doi:10.1186/s12864-021-08075-2)
Supplement: Supplementary file 7 — Additional file 7: Table S4. Median, mean, and standard deviation of the number of CNVs per animal, referred to as count, and the length of CNVs within breed. [file 12864_2021_8075_MOESM7_ESM.docx]

Table S4: Median, mean, and standard deviation of the number of CNVs per animal, referred to as count, and the length of CNVs within breed.

| Breed | Median - count | Median - length (kb) | Mean - count | Mean - length (kb) | Standard deviation - count | Standard deviation - length (kb) |
| --- | --- | --- | --- | --- | --- | --- |
| Charolais | 24 | 23.2 | 29.8 | 52.4 | 19.3 | 106.7 |
| Holstein-Friesian | 75 | 19.1 | 113.1 | 37.9 | 99.0 | 73.0 |
| Limousin | 23 | 23.4 | 27.2 | 47.0 | 18.0 | 104.5 |
